# Feasibility, challenges, and future prospects of microalgae-based bioremediation technique for removing microplastics from wastewater

**DOI:** 10.3389/fbioe.2023.1288439

**Published:** 2023-10-19

**Authors:** Ning Gao, Ruoxu Ning, Xiangyuan Deng

**Affiliations:** ^1^ Key Laboratory of Ecological Impacts of Hydraulic-projects and Restoration of Aquatic Ecosystem of Ministry of Water Resources, Institute of Hydroecology, Ministry of Water Resources and Chinese Academy of Sciences, Wuhan, China; ^2^ Huangdao Gaoning Clinic of Integrated Traditional Chinese and Western Medicine, Qingdao, China; ^3^ College of Biotechnology, Jiangsu University of Science and Technology, Zhenjiang, China

**Keywords:** microplastics, microalgae, wastewater, bioremediation technique, feasibility, challenges

## 1 Introduction

Microplastics are plastic particles and fragments with a size of less than 5 mm (Thompson et al., 2009). Depending on the source, they are classified as primary and secondary microplastics. Sources of primary microplastics are household sewage discharge, including polymeric particles from cosmetic beads, cleaning products, feedstocks used to make plastic products, and plastic pellets or powders used for air blasting (Hamidian et al., 2021). Sources of secondary microplastics are the degradation of macroplastics through UV radiation, mechanical erosion, and biologic analysis (Liu et al., 2019). Recently, microplastics have been detected in various environments, and recognized as emerging pollutants due to their negative effects on both the ecosystem and public health (Jeong et al., 2023). Previous studies have documented that wastewater treatment plants are a primary recipient of terrestrial microplastics, and abundances of microplastics in wastewater range from 0.28 particles L^−1^ to 3.14 × 10^4^ particles L^−1^ (mean value: 1.90 × 10^3^ particles L^−1^; median value: 57.60 particles L^−1^) (Liu et al., 2021). Moreover, wastewater plays an important role in the linkage between microplastics and natural waterbodies (Bakaraki Turan et al., 2021). Therefore, how to remove microplastics from wastewater has received extensive attention among researchers.

It has been reported that microplastics can be removed during wastewater treatment processes of primary (e.g., coarse and fine screening, grit and grease elimination, skimming, and primary sedimentation), secondary (e.g., activated sludge related process, biofilm related process, membrane bioreactor process, and granular sludge process), and tertiary (e.g., coagulation, sand filtration, membrane filtration, adsorption, denitrification/nitrification filtration tank, and ozone oxidation) (Zhang et al., 2020; Hamidian et al., 2021). In existing wastewater treatment plants, removal efficiency of microplastics is often less than 50% (Xu et al., 2021), although it could reach to 91.7% and 99.9% in the plants of Canada and Finland, respectively (Talvitie et al., 2017; Gies et al., 2018). These data indicate that the current removal techniques of microplastics are not very efficient, and the differences in removal efficiency may be caused by the applied methods used in each wastewater treatment plants (Xu et al., 2021). In addition, it must be noted that specific processes for microplastic removal have not been designed in existing treatment plants because microplastics are emerging pollutants in wastewater (Ngo et al., 2019).

Recently, microalgae-based bioremediation technique has been proved to be a bioalternative strategy for removing microplastics from wastewater. For example, multiple kinds of microplastics could be removed effectively by microalgae (*Scenedesmus abundans*) via hetero-aggregation (Cheng and Wang, 2022). Moreover, microplastics could also be removed by *Cyanothece* sp., *Tetraselmis* sp., and *Gloeocapsa* sp. through different mechanisms (Lu et al., 2023). But the technique still has a long way to reach practical application due to some key constraints, such as long retention time, toxicity of microplastics to microalgae, and environmental risk of residual algal cells. This paper reviewed the feasibility, challenges, and future prospects of the technique used for removing microplastics from wastewater, and put forward some opinions to encourage researchers for discovering practically-feasible solutions to the key limiting factors.

## 2 Feasibility, challenges, and future perspectives

### 2.1 Feasibility of using microalgae to remove microplastics from wastewater

Recently, some investigations have been performed on the feasibility of using microalgae-based bioremediation technique to remove microplastics from wastewater, and corresponding results document that this technique has a wide range of advantages, such as highly effective removal of microplastics, reduction of energy requirement and operational cost, recovery of nutrients in wastewater, and low risk of microplastics to the environment, relative to the conventional methods (Cheng and Wang, 2022). For example, polystyrene, polylactide, and poly (methyl methacrylate) microparticles could be removed effectively by *Scenedesmus abundans* with the highest removal efficiencies of 84, 87, and 98%, respectively (Cheng and Wang, 2022). Possible mechanisms underlying the removal of microplastics by microalgae have been investigated, which are summarized as follows: 1) microalgae could generate extracellular polymers for aggregating and flocculating microplastics (Cunha et al., 2019; Cunha et al., 2020); 2) microalgae could break down the polymer matrix into simpler monomers (Reddy and Nair, 2022); and 3) electrostatic charge onto algal surfaces could interact with that on the microplastic particles (Lu et al., 2023). Thus, the microalgae-based bioremediation technique has been considered as a promising bioalternative strategy for mitigating emerging microplastics contamination (Krishnan et al., 2023). In addition, microalgae could uptake and utilize nutrients in wastewater for nutrient removal and biomass production, and the harvested algal biomass could be used as a feedstock for producing biofuels and other value-added biochemicals (e.g., proteins, pigments, vitamins, and carbohydrates), which would benefit the economy of microplastic removal by microalgae (Deng et al., 2018; Knoshaug et al., 2018).

### 2.2 Challenges of the microalgae-based bioremediation technique

Relative to conventional methods, microalgae-based bioremediation technique has superiority on the removal of microplastics from wastewater, but it still faces some main challenges restricting its large-scale application, which are concluded as follows.

(1) Long retention time: Relative to natural waters, wastewater could alter the properties of microplastics effectively due to the high levels of metals, organic pollutants, pharmaceuticals, personal care products, and microorganisms (Hamidian et al., 2021). Thus, microplastics in wastewater are found to possess different properties, such as shape, size, density, surface charge, and mobility, which play an important role in influencing the interaction of microplastics and microalgae (Hamidian et al., 2021; Parashar and Hait, 2023). Because of these properties, they are extremely difficult to be removed only by microorganisms over a short period of time. It has been reported that the hydraulic retention time in a typical biological reactor is in the range of 4–12 h, which could not meet the requirements of rapid treatment of microplastics from wastewater (Zhang et al., 2020). Therefore, microalgae-based bioremediation technique is not ideal for removing microplastics from wastewater due to the long retention time.
(2) Toxicity of microplastics to microalgae: As the main recipients of terrestrial microplastics, their abundances in wastewater are significantly higher than that in other environments (Liu et al., 2021). Moreover, microplastics could absorb other environmental pollutants (e.g., heavy metals, antibiotics, personal care products, and fuel aromatics) in wastewater, and exude some additives, such as bisphenol A, dibutyl phthalate, and diethylhexyl phthalate into wastewater (Song et al., 2020; Rahman et al., 2023). Previous studies have documented that microplastics could cause toxicity to microalgae population by reducing photosynthetic efficiency and increasing reactive oxygen species production (Dong et al., 2022). The toxicity could influence the removal efficiency of microalgae-based bioremediation technique for removing microplastics from wastewater. Thus, toxicity of microplastics to microalgae should not be ignored when this technique is used to remove microplastics from wastewater.
(3) Environmental risk of residual algal cells in the treatment system: Until now, the frequently-used methods for microalgae harvest are sedimentation, flotation, centrifugation, and filtration, whose recovery efficiencies are 10–90, 50–90, >90, and 70%–90%, respectively (Liu et al., 2023). Some algal cells would remain in the treatment system, which may pose a potential risk to environment because they could cause a negative impact on the diversity of aquatic ecosystems (Padervand et al., 2020). Thus, it is very important to harvest algal cells completely from the treatment system when microalgae-based bioremediation technique is used to remove microplastics from wastewater.

### 2.3 Future prospects of the microalgae-based bioremediation technique

In order to overcome the above challenges, future investigations should be carried out in the following aspects. Firstly, occurrence, characteristic, abundance, and fate of microplastics in wastewater should continue to be investigated for obtaining their basic data. Secondly, the retention time should be further shortened by different strategies, such as selection of microalgae species, optimization of operational conditions, development of novel treatment system, and integration of the technique with other conventional physical and chemical methods. Thirdly, toxic mechanism of microplastics against microalgae should be revealed ulteriorly, which could help to understand the risks of microplastics to aquatic ecosystem and the interaction performance between microplastics and microalgae. Finally, effective harvest methods (e.g., fungal-induced flocculation and magnetic flocculation) should be established in order to prevent the potential environmental risk of residual algal cells in the treatment system.

## 3 Summary and recommendations

**FIGURE 1:**
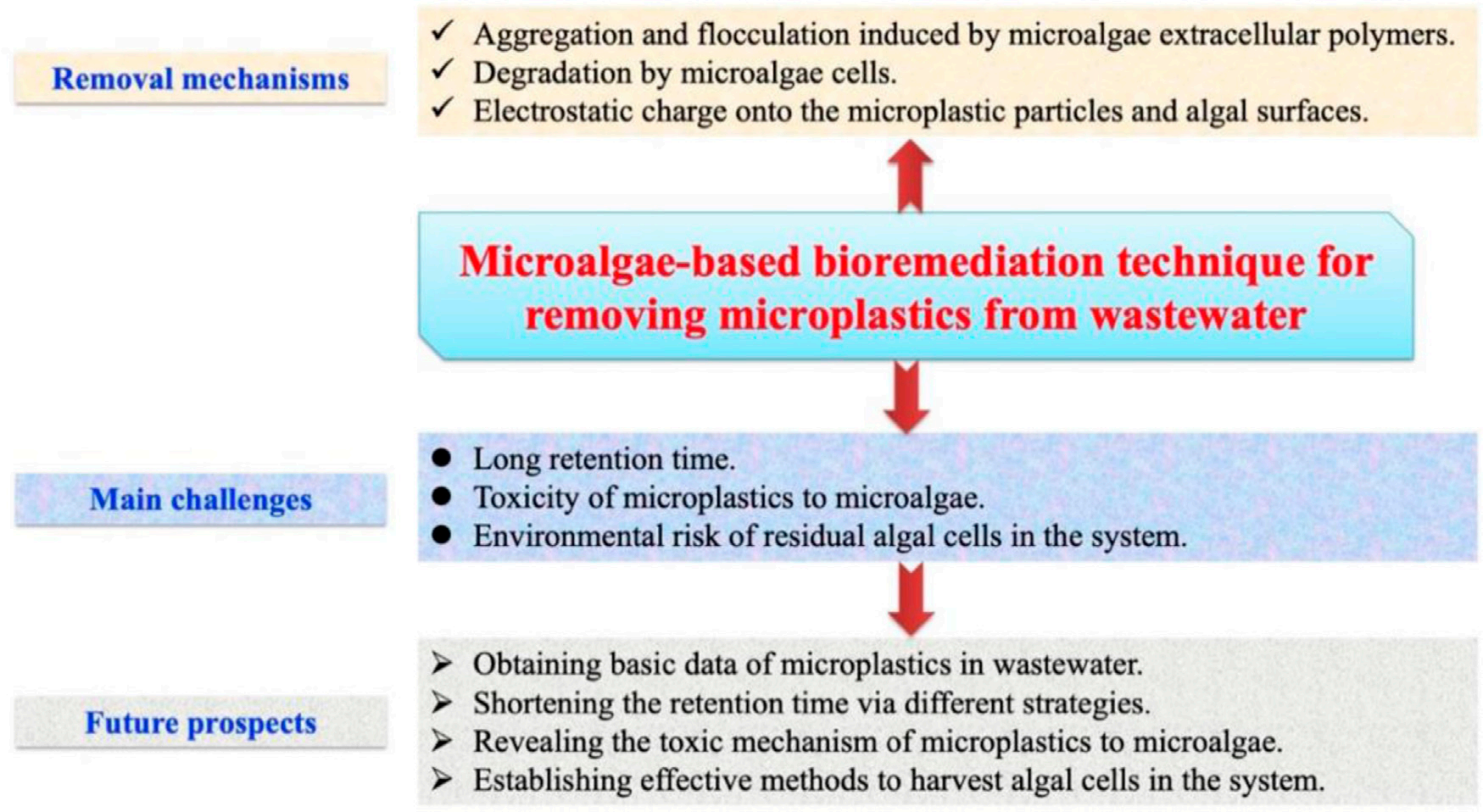
Removal mechanisms, main challenges, and future prospects of microalgae-based bioremediation technique for removing microplastics from wastewater.

On the basis of our research experiences and literature reviews, it is concluded that using microalgae-based bioremediation technique to remove microplastics from wastewater is entirely feasible, but it faces some challenges limiting its practical application (Figure 1). The main challenges are long retention time, toxicity of microplastics to microalgae, and environmental risk of residual algal cells. In response to these challenges, some potential solutions have been proposed in this paper, which may provide research topics to investigators in this field.
